# Neurosurgical Theatres’ Carbon Net Efficiency: A Service Improvement Project Conducted via the Oxford Cranioplasty Pathway

**DOI:** 10.3390/healthcare14131828

**Published:** 2026-06-24

**Authors:** Sara Lonigro, Yaw Antwi-Yeboah, Francesca Carella, Tania dos Reis, Gregory P. L. Thomas, Rosanna Ching, Lara Prisco, Mario Ganau

**Affiliations:** 1 Nuffield Department of Clinical Neurosciences, University of Oxford, Oxford OX3 9DU, UK; 2 Department of Neurosurgery, Oxford University Hospitals NHS FT, Oxford OX3 9DU, UK; 3 Department of Plastic and Reconstructive Surgery, Oxford University Hospitals NHS FT, Oxford OX3 9DU, UK; 4 Oxford Cranioplasty Pathway, Oxford University Hospitals NHS FT, Oxford OX3 9DU, UK; 5 Department of Anaesthesia, Oxford University Hospitals NHS FT, Oxford OX3 9DU, UK; 6 School of Medicine, BAU International University Batumi, 6010 Batumi, Georgia

**Keywords:** calvarial reconstruction, surgical pathway, carbon net efficiency

## Abstract

**Highlights:**

**Public health relevance—How does this work relate to a public health issue?**

- Reducing carbon emissions from health and care activities aligns with the goals set by the United Nations Climate Change Conference of Parties (UN-CC-COP).

**Public health significance—Why is this work of significance to public health?**

- The service improvement project described in this manuscript demonstrates the effectiveness of a partnership between public institutions and medtech companies.
- Material choices, which are relevant in cranioplasty surgery, can also influence the carbon efficiency of cranioplasty pathways without compromising patients’ safety.
- Public health implications—What are the key implications or messages for practitioners, policy makers and/or researchers in public health?
- The diverse needs of various surgical subspecialties call for an ad hoc review of interventions aimed at reducing the carbon footprint related to surgical procedures.
- Different stakeholders bring valuable perspectives on designing service improvement projects to achieve carbon net efficiency of neurosurgical theatres.

**Abstract:**

Background: The research question explored in this study revolves around the quantitative evaluation of the carbon footprint of cranioplasty surgery, a neurosurgical intervention meant to reconstruct skull defects. Methods: Following a calculation of the emissions pertaining to Scope 1 to 3 of the Greenhouse Gas (GHG) Protocol, the authors engaged with various stakeholders to identify possible interventions meant to drive the carbon efficiency of a cranioplasty pathway. The service improvement project (SIP) that ensued was aimed at reducing the volume and weight of the packaging materials for cranioplasty shipping boxes, and decreasing the paper consumption relative to the preparation of user manuals without compromising patients’ safety. Results: Our analysis indicates a cumulative carbon footprint of 104.35 kg CO_2_e for a single unilateral cranioplasty operation, where packaging corresponds to 6.4% of Scope 3 emissions and 1.41% of its total emissions. Of note, our SIP led to an overall 76.53% decrease in the number of emissions generated by the packaging equivalent required for a unilateral titanium implant. Conclusions: This study demonstrates the effectiveness of a partnership between public institutions and medtech companies in driving carbon net efficiency of a cranioplasty pathway, and we suggest that such approach is scalable to other surgical specialties.

## 1. Introduction

Human driven climate change poses a significant risk to both planetary and public health through extreme weather events driving air pollution, transmission of climate-dependent pathogens alongside food and water scarcity [1].

The associated health inequalities arising from the adverse impact of global warming prompted over 70 countries at the latest United Nation Climate Change Conference of Parties meetings (COP 26 to 30) to address the need of reducing the carbon footprint of primary emitters, including health and care services across the globe (https://unfccc.int/process/bodies/supreme-bodies/conference-of-the-parties-cop accessed on 20 December 2025). In England, the National Health Service (NHS) accounts for 4–5% of the total national carbon footprint, equivalent to a staggering 25% of public sector emissions [2,3,4,5]. As a result, the NHS has committed to reduce its emissions across each scope, as defined by the Greenhouse Gas (GHG) Protocol, aiming to deliver a carbon neutral service by 2040. Recent estimations predicted the need to dissipate 6.1 MtCO_2_e from the NHS Carbon Footprint (emissions controlled directly) and 24.9 MtCO_2_e from the NHS Carbon Footprint Plus (indirect emissions which the organization can influence) for the NHS to achieve its ambitious target. Among the initiatives created to support this ambitious goal, a notable educational one has been designed by the NHS Leadership Academy, which started offering a blended as well as a fully virtual version of the Sustainability Leadership for Greener Health and Care Programme since 2023 (https://www.leadershipacademy.nhs.uk/programmes/leading-for-sustainable-health-and-care-programme/ accessed on 20 December 2025).

In modern hospitals, various activities compete to ensure quality of care and patients’ safety, while driving higher productivity; nevertheless, also carbon net efficiency contributes to this equation and has been included in the “triple bottom line” framework suggested by Elkington in 1994 [6]. More specifically, operating theatres represent one of the areas with greatest potential for improvement due to their resource-intensive nature, resulting in a disproportionate environmental impact, estimated to be three to six times higher in comparison to other clinical areas [1,7,8]. While the energy demands required to preserve optimal theatre conditions and to operate sophisticated surgical equipment, alongside the use of anesthetic gases, pharmaceuticals and single-use products constitute the major sources of direct and indirect emissions associated with surgical care [9], evaluating the carbon footprint of other components and processes underpinning surgical operations constitutes a fundamental step in identifying key carbon hotspots, providing the foundation for the development of strategies aimed at safely reducing the environmental impact of surgery [10].

**Figure 1:**
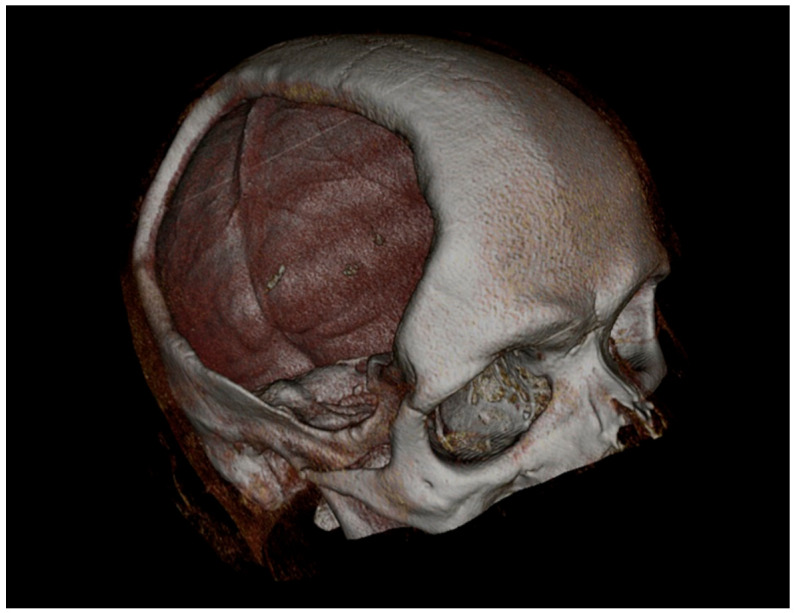
3D rendering of a non-contrast CT head performed for cranioplasty designing purposes. At the Oxford Cranioplasty Pathway all calvarial defects secondary to decompressive craniectomies are studied with CT images acquired using a dedicated cranioplasty protocol (Siemens SOMATOM Drive): 120 kV and 300 mAs with automated tube current modulation (CARE Dose4D). The 3D models are generated from 0.5 to 0.7 mm thin-slice reconstructions to accurately visualize the bony defect (Kernel Hr60) and surrounding soft tissue contours (Kernel Hr38), whereas brain imaging is obtained with conventional 5 mm thin-slice reconstructions (Kernel Hr38).

Currently, the literature offers a limited set of studies where the carbon footprint of common surgical procedures has been estimated, nonetheless this seems to lie within a range of 6–814 kg for a single operation [1,11,12]. Such estimations are fairly specific to procedures in the field of general surgery, orthopedics and ophthalmic surgery; therefore, a fundamental gap in the existing literature regarding other surgical specialties exists and this requires further investigations. Leveraging on the pioneering work on the environmental footprint of neurosurgery from Talibi et al. [7], our study aims to extend said analysis by offering a first-time quantitative evaluation of the carbon footprint of a cranioplasty pathway. Cranioplasty is a neurosurgical intervention meant to provide calvarial reconstruction following decompressive craniectomy (Figure 1), a life-saving procedure offered for the medical refractory management of traumatic brain injury, tumor resection, cerebrovascular event or intracranial infection [13,14].

Restoration of skull integrity, alongside improved neurological function, is afforded through the implant of an autologous bone flap or, more commonly, an artificial, custom-made prosthesis. In line with the Human Tissue Act (www.hta.gov.uk accessed on 20 December 2025), regulating activities concerning the removal, storage, use and disposal of human tissue, the current standard of care in the United Kingdom favors the provision of custom-made implants designed to precisely complement the anatomical features of the calvarial defect over autologous bone flaps.

**Figure 2:**
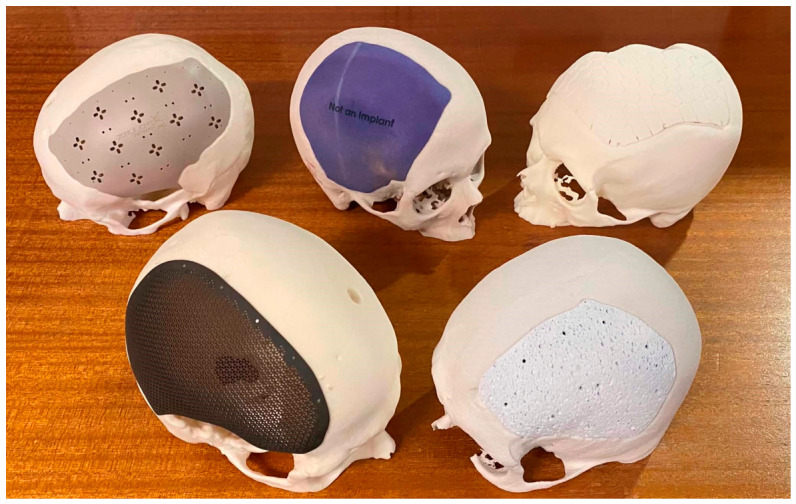
3D replicas of cranioplasty plates and models of calvarial defects used in our outpatients’ consultations to discuss the pros and cons of various materials and engage patients whenever feasible and appropriate in the decision-making regarding product selection. (**Top row**) PEEK cranioplasty, PMMA/PEKK model, hybrid titanium–calcium phosphate model. (**Bottom row**) Titanium cranioplasty, PHA cranioplasty.

Numerous materials, ranging from medical-grade titanium (Ti6Al4V), polymers (such as polymethylmethacrylate, PMMA, polyetherketoneketone PEKK, polyetheretherketone, PEEK), and bio-mimetic ceramics (such as porous hydroxyapatite, PHA), are available in the cranioplasty market [13,15,16] and most of them are routinely used in our service (Figure 2).

To address our research question and quantitative evaluation of the carbon footprint of a cranioplasty pathway, we present an analysis of the external environment specific to the field of cranioplasty surgery, and an action plan devised to safely improve the sustainability of our surgical pathway, aiming to mitigate the environmental burden arising from the delivery of this service at the John Radcliffe Hospital (Oxford University Hospitals NHS Foundation Trust).

## 2. Materials and Methods

This study falls under the umbrella of CREPES (Calvarial REconstruction Pathway: an Exploratory Study): an over-arching European study group exploring the impact of structured cranioplasty pathways (standard operating procedures for referral processes, decision-making and follow up strategies for patients undergoing cranioplasty surgery) on patient outcomes. CREPES protocol (originally submitted to the European Association of Neurosurgical Societies research committee in 2023) includes multiple research projects; however, this is the very first one entirely dedicated to the sustainability of cranioplasty surgery.

### 2.1. Location

The Department of Neurosurgery is located in the newly constructed West Wing of the John Radcliffe Hospital in Oxford (United Kingdom), a large quaternary-care center holding 24 surgical suites across two hospital sites. The surgical suite consists of an operating theatre with separate anesthetics and washing rooms, alongside an additional area for trolley preparation. The following design is responsible for the high operative volume, estimated at 30,000 cases per annum [8]. Of these, 41 cranioplasty procedures are performed yearly on average (range 39–63/yearly over the past 8 years), making the Oxford Cranioplasty Pathway the third center per operative volumes according to the first report of the United Kingdom and Ireland Cranioplasty Registry [13].

### 2.2. Carbon Footprint Estimation for Cranioplasty Surgery

The methodology of this study focuses on assessing the environmental impact of a single cranioplasty operation by estimating its associated carbon footprint. To standardize the assessment, only unilateral cranioplasty have been considered (this is because for bi-frontal defects some companies offer a single plate whereas others offer two assembling plates in a single shipping box or in two separate shipping boxes). Evaluating the carbon footprint of a process, such as a surgical operation, allows hospitals to quantify the contribution of each source of direct and indirect GHG emissions underpinning the process. In this study, calculation of the carbon footprint conforms with the principles illustrated within the ISO (International Organization for Standardization) 14,064 bill, an international standard providing guidance on the quantification and reporting of GHG emissions at the organizational level, in line with the GHG Protocol [17,18]. According to this standard, emissions can be classified into three scopes:

- Scope 1: GHG emissions produced onsite by owned or directly controlled sources within an organization. These include fossil fuel combustion and use of anesthetic gases.
- Scope 2: Indirect sources of GHG emissions associated with the use of purchased energy including electricity, heating, steaming and cooling.
- Scope 3: Remaining indirect sources of GHG emissions within an organization including the supply chain required to produce pharmaceuticals and medical equipment, travel undertaken by members of staff, patients or visitors alongside water supply and waste disposal.

Once source of emissions for a single unilateral cranioplasty operation were identified and sorted into the respective scopes, the “bottom-up” model was applied as the preferred methodology for carbon footprint estimation [1]. The “bottom up” approach is a process-based method which provides a highly specific quantitative analysis of the carbon footprint underpinning a process through the collection of activity data and their corresponding emissions factors [1]. Activity data are intended as any source of process-specific activity which generates GHG emissions, such as electricity use [19]. Emission factors are coefficients, derived from the life cycle assessment (LCA) of raw materials, which are used to translate activity data into emission values [19]. The accuracy of these two variables is crucial to yield reliable results. Emission factor data were gathered from a variety of published resources which included the Inventory of Carbon and Energy (ICE) and the UK governmental Department for Environment, Food and Rural Affairs (DEFRA) databases, alongside data derived from previously conducted studies available in the literature [8,10,20,21]. As variability in emission factor reporting was encountered across different sources, this study prioritized the use of a single database (https://www.climatiq.io/data accessed on 20 December 2025) to ensure consistency in the results, and we considered the use of alternative sources from the literature only in the instance of processes or materials where emission factors were not reported in the primary database. Subsequently, the following formula previously validated by Misrai et al. [19] was employed to determine the carbon footprint of each product and process involved in a single cranioplasty operation:

*Carbon footprint* (kg CO_2_e) = *activity data* × *emission factor*

While emission factors are calculated via LCA, which quantify the carbon cost to extract raw materials, manufacture them, and ship the final product to the hospital; applying the carbon footprint formula to hospital environments, in order to accurately calculate GHG arising from clinical or surgical practice, requires breaking down individual activity data (e.g., those relative to a single operation) into measurable parts. More specifically the carbon footprint of a surgical intervention can be calculated considering three main sources of GHG: Anesthetic Gases, Surgical Consumables, and Theatre Energy. Those three ‘hotspots’ reflect the emissions’ classification mentioned above: Chemical Impact (a.k.a. Scope 1), Supply Chain Impact (a.k.a. Scope 2) and Facility Impact (a.k.a. Scope 3). Healthcare-related organizational activities mainly produce carbon dioxide (CO_2_), methane (CH_4_), nitrous oxide (N_2_O) and fluorine-containing gases [3,22]. As CO_2_ constitutes up to 85% of healthcare-related emissions, it is regarded as a reference gas for the purpose of converting emissions of non-CO_2_ GHG into carbon dioxide equivalents (CO_2_e), providing a common unit of measure when calculating the total carbon footprint of a process [1,19].

Following a definition of the goal of this study, three additional steps were undertaken to complete our assessment of the carbon footprint of cranioplasty surgery: in the surgical arena: (a) identifying which resources are key to this surgery, (b) measuring the resource utilization and (c) attributing a footprint to those resources. For clarity, step (a) required a qualitative approach with the definition of our constraints (e.g., ability to track the entire supply chain for each resource/activity), whereas step (b) required a quantitative approach and the creation of an inventory (e.g., in the instance where activity data, in our study, comprised non-CO_2_ GHG emissions, the above product was multiplied by a gas-specific Global Warming Potential (GWP100) value, accounting for differences in impact on global warming, exerted by a wide spectrum of GHGs, relative to CO_2_). The final step (c) consisted in measuring the entire environmental impact of cranioplasty surgery and required the analysis of the total carbon footprint by adding up each individual GHG contribution (Scope 1-2-3) for every product and process underpinning this neurosurgical intervention (see Table S1 from Supplementary Material comprehensive of steps a-b-c).

#### 2.2.1. Scope 1

No products or processes relative to Scope 1 of the GHG protocol were included in the carbon footprint analysis conducted in this study. The primary reason was due to the hospital’s preferential choice of administering intravenous anesthetics over volatile gases, such as sevoflurane, isoflurane or desflurane, for induction of anesthesia in preparation for cranioplasty surgery.

#### 2.2.2. Scope 2

Data highlighting the energy requirements for the provision of heating, ventilation, cooling (HVAC) and lighting during a cranioplasty operation were derived from the work of MacNeill and colleagues [8]. According to this study, the surgical suites at the John Radcliffe Hospital employed a single duct reheat system utilizing purchased natural gas and electricity as the primary sources of fuel to meet the facility’s HVAC demands; such approach is very ubiquitous across the NHS [8]. Since the quantity of energy with its associated GHG emissions were expressed as yearly amounts, the data were manipulated in order to provide an approximate value applicable to the energy consumed in the surgical suite during a single cranioplasty surgery session [8]. Such calculation was based on the estimation that each surgical procedure to implant a unilateral custom-made cranioplasty plate requires an average of 4 h of operative time (range 2 h to 5 h, from induction in the anesthetic room to transfer to the recovery suite). As a result, annual energy amounts in MWh/year were converted into hourly figures expressed in kW consumed in one hour of operative time. These activity data were multiplied by their respective emission factors, as specificized by DEFRA for electricity and natural gas purchased in the UK [20].

#### 2.2.3. Scope 3

Most of the carbon footprint analysis performed in our study focused on covering in detail sources of emissions within Scope 3. Firstly, the surgical pathway underpinning a cranioplasty operation was scrutinized to identify the principal products and processes involved. These were, then, grouped under broad categories such as medical equipment, non-volatile pharmaceuticals, non-volatile chemicals and waste disposal [9]. For the purpose of this study, the activity data under medical equipment included the cranioplasty implant, surgical instruments and devices used in the procedure, surgical drapes and protective personal equipment (PPE) [23]. Packaging and user manual data were included in our analysis where accessible. Manufacturer information provided datapoints on the weight and material composition characterizing the prosthesis and its packaging which included a 3D model of the calvarium and a surgical guide for planning the operative approach.

The carbon footprint of further surgical devices, reusable instrument sets and single-use items (see Table S1 from Supplementary Material for details of surgical sets considered) was determined using activity data extrapolated from the work of Rizan and colleagues [10]. Emission coefficients for non-volatile pharmaceuticals and chemicals were obtained from previous studies conducted by Parvatker et al. and Rizan et al. [10,22,24].

### 2.3. Carbon Footprint of Various Cranioplasty Materials

The same formula employed to determine the carbon footprint of each product and process involved in a single cranioplasty operation was used to determine the different carbon footprint of various cranioplasty materials. The estimated carbon footprint of various custom-made cranioplasty plates was obtained by calculating the average weight of unilateral cranioplasty plates from our own surgical series, whenever possible those figures were double checked with the producing companies (of note the company producing a hybrid titanium/calcium phosphate cranioplasty discontinued manufacturing during this study). A typical cranioplasty plate was considered as a unilateral plate meant to cover a defect with a diameter measuring 13.5 cm and an area of ~100 cm^3^ [13,14,15,16]. Similarly to the approach taken to calculate the carbon footprint of cranioplasty surgery, emission factors considered in the calculation were extracted from https://www.climatiq.io/data (accessed on 20 December 2025). the largest open source, free database of vetted emission factors and verified on the existing scientific literature for confirmation.

### 2.4. Designing the Sustainability Plan

The development of a sustainability plan for the Oxford Cranioplasty Pathway was based on the multi-disciplinary contributions of various stakeholders involved in this surgical pathway. According to Miles’ theory, stakeholders were categorized as *Influencers*, *Claimants*, *Collaborators* and *Recipients*, in relation to their capacity to influence the provision of this service [25]. *Influencers* included hospital managers; *Claimants* included surgeons, medical and nursing staff; *Collaborators* included coordinators of the cranioplasty service, representatives of suppliers and manufacturers; and *Recipients* included patients and their next of kin. Each aspect of the cranioplasty pathway was analyzed and argued for improvement at several stakeholders’ meetings held between August 2023 and the Spring of 2024. Engaging with several cranioplasty suppliers and the other stakeholders led to the suggestion that exploring ways to reduce the packaging of cranioplasty boxes without compromising their safety was the most sensible approach. Along this process we set various SMART goals (Specific, Measurable, Achievable, Relevant, Time-bound), identifying actionable steps (e.g., increasing efficiency, boosting surgical productivity, reducing waiting times, reducing surgical waste) and we committed to regular reporting of our progresses. Given the growing interest in producing greener products, some cranioplasty companies serving the Oxford Cranioplasty Pathway expressed their interest in collaborating with us on one of those SMART goals: accordingly we designed together a service improvement project (SIP) around the objective of reducing surgical waste and we specifically attempted to address the critical issue of packaging waste associated with the high volume in our calvarial reconstruction activity. In fact, this was seen as a mutually beneficial endeavor: on one hand, this SIP had the potential to reduce the costs associated with production and shipping of the cranioplasties, on the other hand, any viable solution would have reduced the amount of clinical waste produced in our operating theatres. Once the research question and protocol were finalized, this SIP was registered on Ulysses, the electronic governance system of Oxford University Hospitals NHS FT (Registration Number 9284) and formally approved in November 2024. Xilloc Medical B.V., Maastricht, Netherlands, one of the companies producing titanium and PEEK patient specific implants, offered to partner with us in this SIP. Given that titanium has emerged as the most common choice for calvarial reconstruction in the UK [13], we elected to focus our attention on a typical titanium cranioplasty shipping box. As such, our SIP aimed at: (a) reducing the volume and weight of the packaging materials per shipping box, and (b) decreasing paper consumption relative to the preparation of its associated user manual.

## 3. Results

### 3.1. Carbon Footprint Analysis

The quantitative analysis conducted in this study yielded a cumulative carbon footprint of 104.35 kg CO_2_e for a single unilateral cranioplasty operation. Scope 2 emissions accounted for 78% of the total carbon footprint, contributing the most to this total figure with an estimated 81.39 kg CO_2_e released to fulfil the suite’s HVAC requirement of 4858.92 kWh. Emissions within Scope 3 comprised the remaining 22% of the overall carbon burden corresponding to 22.96 kg CO_2_e released in the atmosphere. Within Scope 3, items grouped under medical equipment generated approximately 9.64 kg of carbon dioxide equivalents. Unsurprisingly, disposal of surgical waste radiated the highest amount of CO_2_ in its category, equivalent to 12 kg CO_2_e or 52% of Scope 3 emissions. On the other hand, non-volatile pharmaceuticals and chemicals were responsible for only 1.32 kg CO_2_e (a summary of our calculations is provided in Table 1).

The second part of our quantitative analysis consisted in estimating the differences in terms of carbon footprint among the various materials available for the manufacturing of cranioplasty plates. Our analysis indicates that the most eco-friendly cranioplasty plates are those made of PHA. Such a result is driven by the emission factor for PHA which is the lowest (0.37418 kg CO_2_e) among all emission factors considered. While titanium and PMMA possess fairly comparable carbon footprints, the production of PEEK cranioplasty plates seems to yield the highest consumption (1.49151 kg CO_2_e), likely reflecting the energy-intensive, high-temperature synthesis and complex processing (see Table 2 for average weight of a typical cranioplasty plate, emission factors and the estimated carbon footprint).

### 3.2. Sustainability Plan

Multiple efforts have been made to reduce the quantity of plastic used for wrapping the contents within a cranioplasty box to a minimum; these required a continuous check that the shipping box remained compliant with the existing regulations and quality standards (ISO 11607-1, ISO 11607-2 and EN 868-5). The best way forward was to shift from polyurethane foam cushions to air-filled high-density polyethylene bags, which provided a significantly lower emission factor of 2.08 kg CO_2_e/kg compared to the 4.84 kg CO_2_e/kg released in the life cycle of polyurethane flexible foam [21]. Another advantage offered by such a solution is that polyethylene products can be recycled more easily than polyurethane plastics as the latter require energy-intensive techniques for reprocessing [26].

Furthermore, inspired by the digital transformation in all clinical wards at our hospital we decided to go paperless and we reached an agreement with the cranioplasty manufacturing company to convert the printed user manuals and surgical guides into electronic versions. Only the accompanying stickers required for positive patient identification have remained in paper form. Implementing the above modifications in the supply chain of our cranioplasty pathway has resulted in an overall decrease in the number of emissions generated by the packaging equivalent of a titanium cranioplasty box to 76.53% (see Table 3 for the calculations made before and after this SIP). Such results are in keeping with the goal set for NHS providers to achieve clinical waste segregation targets of 20:20:60 HTI (high-temperature incineration), AT (alternative treatment) and OW (offensive waste) by 2026 (https://www.england.nhs.uk/long-read/nhs-clinical-waste-strategy/ accessed on 20 December 2025).

## 4. Discussion

Carbon footprinting studies in surgical healthcare are a rapidly emerging research field, driven by the fact that hospitals account for approximately 5% of GHG emissions, with the weight of operating theatres being 3–6 folds higher than any other clinical and non-clinical area. The focus from regulatory bodies and healthcare services has fostered a renewed interest in expanding our understanding of the drivers of carbon efficiency and the currently unexploited opportunities for the improvement of hospitals’ performances. Presently, the literature offers limited evidence regarding the ecological impact of procedures across a range of surgical specialties. Findings derived from previous systematic reviews showed that a single operation can emit as little as 6 kg CO_2_e, in the instance of cataract surgery, and up to 814 kg CO_2_e in more complex cases such as in a robotic hysterectomy [11,12,27,28]. In the field of neurosurgery, only one study has evaluated the average emissions per neurosurgical case estimated at 24.5 kg CO_2_e [7]. However, emissions associated with theatre energy consumption, single-use products and cranial implants were excluded from the cumulative carbon footprint proposed in the above investigation. Our study has attempted to move forward from the initial work by Talibi et al. and, as such, the carbon footprint analysis conducted in this study offers a first-time insight into the environmental burden of a single cranioplasty operation (equivalent to 104.35 kg CO_2_e). Purchased electricity, medical equipment, waste processing, non-volatile pharmaceuticals and chemicals comprised the principal sources of GHG emissions evaluated in our study. In particular, disposal of surgical waste radiated the second highest quantity of CO_2_, most likely due to the considerable use of disposable items and the extensive packaging employed to ship cranioplasty implants. Currently, the literature highlights that waste generated in the operating room from the use of disposable items, including instrument and product packaging, is a significant contributor to the carbon footprint of surgical care. Within this newly emerging body of work, we identified three broad categories of SIPs: audit-only studies, which quantify the carbon burden of waste; resource-optimization studies, redesigning the content of single-use packs or instrument sets to remove unnecessary items; and waste management studies, focusing on optimizing collection, sorting and disposal of surgical waste (Table 4) [29,30,31,32,33,34,35].

Several studies have focused on auditing their local service alone. For instance, Lee YK et al. quantified the packaging waste generated during thyroidectomy, a commonly performed ear, nose and throat (ENT) procedure in the NHS, to estimate a national carbon burden of 1048 kg CO_2_e per year [30]. In the United States, Bravo et al. demonstrated that up to one quarter of disposable items, contained within surgical packs for hand surgery, was unused accounting for a total of 441 kg CO_2_e emitted equivalent to 1650 miles driven in an average petrol car [29]. Despite efforts to quantify emissions associated with surgical services, a few studies have moved beyond to assess the effectiveness of sustainability interventions in their own clinical setting. By revising the contents of a laparoscopic appendicectomy set, Labib et al. demonstrated how targeted sustainability interventions within a service can achieve significant carbon-footprint reductions, as high as 62% less emissions per case [32]. Similarly, our SIP is broadly aligned with the interventions proposed in the current resource-optimization studies as we sought to improve packaging volume and remove non-essential items, such as the user manual, without compromising safety in delivering our service. However, our work adds to the current literature as it sits at the intersection between resource optimization and waste management studies, a much less explored research area with a limited set of comparable studies available.

As in our study, many of the successful projects highlighted in Table 4 have consistently relied on the involvement of frontline staff as key stakeholders. Surgeons, scrub teams and theatre staff bring a more profound understanding of the service to the table, making them well placed to identify wasteful practices and suggest workable improvements. In a recent review, Gorgun et al. showed that 55–97% of surgical staff were willing to change their practice to support sustainability [36]. On the other hand, poor education and weak leadership were amongst the most common barriers to progress. As a result, staff engagement is not simply helpful but essential to drive sustainable change [37].

Across our stakeholder meetings, staff repeatedly pointed out the need for SIP to explore the feasibility of reducing packaging while maintaining the highest standard of manufacturing. In this sense, our SIP was extremely successful because the partnership with Xilloc demonstrated that an effective collaboration can lead to a remarkable decrease in carbon emissions without compromising quality of care.

While our data specifically apply to the shipping of a titanium cranioplasty, a good degree of scalability can be foreseen for other materials too. For instance, PEEK and PMMA cranioplasty would have similar profiles in terms of excellent rigidity, high tensile strength, and creep resistance. On the contrary, our approach would not be suitable for PHA cranioplasties because air-filled high-density polyethylene plastic bags may not be the best packaging solution for such ceramic patient-specific implants, which are notoriously more fragile [15,16]. We therefore recognize the need to consider such aspect when assessing the generalizability of our results. Following on this we shall clarify that, in our SIP, Xilloc outsourced all mechanical stress tests, meant to verify that the solutions proposed by our SIP were actually fit for purpose, to a third-party company dedicated to healthcare packaging (www.nelipak.com accessed on 20 December 2025). Of note, those tests included but were not limited to: transportation, impermeability, falling and aging of the packaging material. Of course, the quality standards ISO 11607-1, ISO 11607-2 and EN 868-5 considered for this SIP and listed in Section 2.4 (Sustainability Plan), which are specific for the European Union and the United Kingdom, may not apply to other regions of the world.

Finally, while our analysis on the carbon footprint associated with various raw materials used in the manufacturing custom-made cranioplasty plates could provide valuable insight for manufacturing companies, it is critically important to recognize that its clinical utility is limited at this stage. Since we firmly believe that each material provides specific pros and cons [38,39,40,41,42], we felt that this aspect of our research should be left outside the scope of our SIP. Nevertheless, few examples of how clinical needs influence material choice for cranioplasty surgery can be useful to the readers. If we consider oncology patients requiring removal of bone flaps due to calvarial erosion caused by primary tumors or metastases [43,44,45], it is important to recognize that they might not be the best candidates for titanium cranioplasties, which could cause imaging artefacts and affect neuroradiological monitoring during follow up. Similarly, the potential need for radiation therapy following custom-made cranioplasty would call for materials that do not heat up, hence ruling out all metals and hybrid cranioplasties [46,47]. Given ongoing investigations to identify alternative materials with more eco-friendly attributes for implantable devices [48,49,50], the market for cranioplasty implants could one day be revolutionized; however, until then the selection of the most appropriate cranioplasty material should be carefully considered by the operating surgeon to meet the clinical needs of individual patients. Obviously, for comparable materials hospitals should consider ‘proximity sourcing’ [51]. Near sourcing is starting to be regarded as a valid alternative to global sourcing in order to leverage supply chain (SC) responsiveness and economic efficiency [52] and can actually enable healthcare organizations to proactively contribute to a more sustainable and environmentally conscious future, while maintaining a focus on quality care for patient well-being [53]. Since logistics may influence the ability of healthcare providers to plan surgical procedures timely, proximity sourcing can bring added value to the management of patients in need of emergency surgery. This is, for instance, the case in patients diagnosed with syndrome of the trephined, where fluctuating neurological deficits secondary to the effect of atmospheric pressure on the brain, can only by stopped by immediately reconstructing the integrity of the skull [54].

### 4.1. Limitations

A few considerations should be taken into account regarding the carbon footprint estimation of a cranioplasty operation proposed in our study. As discussed above, the accuracy of our carbon footprint calculations relies on the precision of the reported emission factors and activity data; for this reason, even if we were unable to conduct a direct LCA for all Oxford cranioplasty plates, we opted for a reliable open source of emission factors, the climatiq.io database, instead. This methodological choice was meant to ensure that all data provided in this study could be verified by other researchers. We also acknowledge that the calculation of those emission factors can be highly specific to production methods, a very good example is the synthesis of PHA which can be generally classified according to the techniques adopted (including wet chemical, dry, thermal, or a combination) [55,56]. These methods are tailored to produce porous structures with varying pore sizes (macro, micro, or mesoporous) depending on the biomedical application, such as scaffold fabrication for bone tissue engineering. Nonetheless, even accounting for different manufacturing processes, which may yield slightly different emission factors compared with the one we adopted in this study, PHA is widely recognized as one of the most biocompatible and eco-friendly material (to the point that the biointegration properties inducing osseointegration and biomimesis represent its greatest advantages compared to all other products) [13,15].

Another limitation of our study, relative to Scope 3, is that we could not track the entire supply chain of various cranioplasties. Other than mentioning the country of manufacturing, we could not provide more specific information regarding the environmental impact of their production and shipping to our neurosurgical center. Developing lean and agile supply chain is critically important to advance an agenda for net-zero healthcare [57,58]; however, as mentioned above such an aspect of hospitals’ daily business also bears potential externalities in terms of optimized clinical outcomes.

Lastly, it is crucial to note that the carbon footprint of very similar surgical operations can vary across studies due to differences in the extent of the study’s scope, methodologies employed, choice of surgical equipment and hospital resourcefulness. For instance, a systematic review found discrepancies in the calculated carbon footprint of cataract surgery where one hospital site reported 81.13 kg CO_2_e whilst the other estimated 151.8 kg CO_2_e for the same procedure [59,60]. Therefore, it is imperative to interpret the carbon footprint reported in our study as an indicative figure, providing a broad idea of the environmental impact of a single unilateral cranioplasty operation.

### 4.2. Future Development

Future studies should anticipate variations in the calculated carbon footprint as a result of the variables mentioned above, recognizing the limited generalizability of carbon footprinting studies. Despite this, the broader aim of our study was to gauge the ecological burden associated with this surgical operation in order to highlight the major carbon hotspots and to propose areas for intervention meant to increase its overall sustainability. The greatest success of our SIP has been to demonstrate that change is attainable even without employing huge financial resources, via a shared collaboration among multiple stakeholders.

Based on the results presented in this study, our team is currently looking at developing new research initiatives. Those include a publication of the full scope of our sustainability plan comprehensive of all other SMART goals and analysis of their health economics implications. The Oxford Cranioplasty Pathway has already proven to be able to provide a threefold improvement in terms of clinical outcomes, resource utilization and surgical productivity; however, creating a long-lasting global impact from local initiatives is always very challenging. As such, we are confident that evaluating our best practices and demonstrating how cost-effectiveness can spur from a reduced environmental impact of our surgical activities is the best way forward to share learning with other cranioplasty services in the UK and overseas.

## 5. Conclusions

Global warming will continue to pose a significant threat to societal well-being until there will be a political and financial will to address it. Since health and care services have been recognized as major contributors to societal GHG emissions, interventions are required to mitigate their environmental impact. This study offers a first-time quantitative analysis of the carbon footprint associated with a single unilateral cranioplasty procedure. Our SIP initiative focused on Scope 3 emissions, and specifically looked at medical equipment and waste disposal, eventually aiming at a consistent reduction in packaging-associated emissions. The 76.53% reduction in the carbon footprint related to packaging only underscores the significant impact of this SIP on our surgical pathway. This result is remarkable, particularly when one considers that packaging corresponds to 6.4% of Scope 3 emissions and 1.41% of total emission relative to cranioplasty surgery. Furthermore, our results indicate that surgical care can be made more environmentally sustainable through collaborative projects. Small contributions, efforts, and savings are all valuable and add up to a significant results over time; for this, the GHG reduction obtained through our SIP multiplied by the annual number of cranioplasty operations performed in Oxford (and all those that have been supported by Xilloc around the world) should inspire newer and bigger sustainability projects extended to many other surgical activities.

## Figures and Tables

**Table 1:** Estimated carbon footprint of cranioplasty surgery as per Scope 2 and 3 emissions.

**SCOPE 2 EMISSIONS**				
**CATEGORIES**	**QUANTITY (kWh/h)**	**CARBON FOOTPRINT** **(kg CO_2_e)**	**CONTRIBUTION TO TOTAL SCOPE 2 CARBON FOOTPRINT (%)**	**CONTRIBUTION TO TOTAL CARBON FOOPRINT (%)**
Heating	132.63	42.44	52	40.67
Ventilation	38.91	21.01	26	20.13
Air Conditioning	24.96	14.73	18	14.12
Lighting	5.96	3.21	4	3.09
**TOTAL (SCOPE 2)**		**81.39**	100	**78.01**
**SCOPE 3 EMISSIONS**				
**CATEGORIES**	**QUANTITY (kg)**	**CARBON FOOTPRINT** **(kg CO_2_e)**	**CONTRIBUTION TO TOTAL SCOPE 3 CARBON FOOTPRINT (%)**	**CONTRIBUTION TO TOTAL CARBON FOOPRINT (%)**
Medical equipment	1.83	9.64	42	9.24
Waste disposal	4.35	12	52	11.5
Non-volatile pharmaceuticals and chemicals	-	1.32	6	1.26
**TOTAL (SCOPE 3)**		**22.96**	100	**22**
**TOTAL CARBON FOOTPRINT**		**104.35**	**100**	**(78.01 + 22)**

**Table 2:** Estimated carbon footprint of custom-made cranioplasty plate. Average weight calculated from our own surgical series and checked with producing companies for a typical cranioplasty plate measuring 13.5 cm and covering an area of ~100 cm^3^. Emission factors considered in the calculation (as per https://www.climatiq.io/data accessed on 20 December 2025): Titanium (20.6 kg CO_2_e/kg); Polymethylmethacrylate (6.37 kg CO_2_e/kg); Polyetheretherketone (17.97 kg CO_2_e/kg); Polyetherketoneketone (22.03 kg CO_2_e/kg); Porous Hydroxyapatite (3.53 kg CO_2_e/kg).

MATERIAL	AVERAGE WEIGHT (g)	ESTIMATED CARBON FOOTPRINT (kg CO_2_e)
3D printed Ti6Al4V **Titanium**	46	0.9476
Polymethylmethacrylate **PMMA**	131	0.8344
Polyetheretherketone **PEEK**	83	1.49151
Polyetherketoneketone **PEKK**	79	1.74037
Porous Hydroxyapatite **PHA**	106	0.37418
**Hybrid Ti6Al4V coated with CaHPO_4_(H_2_O)_2_** Titanium mesh & calcium phosphate	116	Not calculated (*production discontinued in Sept 2023*)

**Table 3:** Estimated change in carbon footprint for packaging used per cranioplasty box before and after this service improvement project (data relative to the cranioplasty plate and craniectomy model were removed from calculations as per methodology section). Emission factors considered in the calculation (as per https://www.climatiq.io/data accessed on 20 December 2025): Polyurethane foam cushions (4.84 kg CO_2_e/kg); air-filled high density polyethylene plastic bags (1.93 kg CO_2_e/kg); Laminated Card User Manual (3.31 kg CO_2_e/kg); Recyclable Double Wall Corrugated Cardboard box (0.7 kg CO_2_e/kg).

	PACKAGING BEFORE S.I.P.	PACKAGING AT RE-AUDIT
**CARBON FOOTPRINT** Polyurethane foam cushions **(kg CO_2_e)**	0.6872	none
**CARBON FOOTPRINT** Air-filled high density polyethylene plastic bags **(kg CO_2_e)**	none	0.0203
**CARBON FOOTPRINT** Laminated Card User Manual **(kg CO_2_e)**	0.4071	None
**CARBON FOOTPRINT** Recyclable Double Wall Corrugated Cardboard box **(kg CO_2_e/kg)**	0.3778	0.3252
**WEIGHT** **Cranoplasty box** **(kg)**	1.12	0.75
**SIZE Cranoplasty box** **Height, Length, Width** **(cm)**	58 × 33 × 11	49 × 26 × 19
**VOLUME Cranoplasty box** **(cm^3^)**	24.206	21.054
**DECREASE IN CARBON FOOTPRINT RELATED TO PACKAGING ONLY** **(%)**	−76.53%	

**Table 4:** Results of comparative analysis showcasing relevant studies tackling Scope 3 emissions broadly categorized into audit, resource optimization and waste management strategies.

CATEGORY	STUDY DETAILS	INTERVENTION	CO_2_e MEASUREMENT	OUTCOME
AUDIT	Bravo et al. [29] United States	Audit of waste generated due to unused disposable supplies in customs packs for hand surgery	Environmentally Extended Input Output (EEIO) Life Cycle Assessment (LCA) model to determine waste associated CO_2_e	22.6% of single-use items opened within a pack constituted waste accounting for a total of 441 kg CO_2_e
	Lee YK et al. [30] United Kingdom	Audit of packaging waste generated locally for thyroidectomy procedures in the NHS to determine national CO_2_e burden per year	Packaging waste from standardized surgical instrument sets and draping was collected for classification, weighing and analysis. Average weights for each waste category were used to estimate the annual weight and CO_2_e impact of packaging-related waste for thyroidectomies in the UK. The Hospital Episodes Statistics (national surgical registry) was employed for the estimation. Carbon footprint was calculated according to the method of waste disposal.	Estimated UK-wide total packaging waste weight: 4.2 tonnes. Estimated UK-wide total packaging waste carbon footprint: 1048 kg CO_2_e
RESOURCE OPTIMIZATION	Chowdhury et al. [31] United Kingdom	Optimization of single-use pack for shoulder surgery to reduce waste and associated carbon footprint	Cradle-to-gate carbon footprint analysis using emission factors provided by the UK Government GHG Conversion Factors for Company Reporting database and the ICE database.	643.8 kg CO_2_e reduction per annum
	Labib et al. [32] United Kingdom	Revision of laparoscopic appendicectomy instrument set	Cradle-to-grave carbon footprint analysis based on emission factors provided by the UK Government GHG factors 2021 report and ICE database.	62% CO_2_e reduction per case. Estimated to reduce by 68% single-use instruments per year equivalent to saving 3 tonnes of CO_2_e emitted during the lifetime of the new set.
	Lee W et al. [33] United States	Instrument tray standardization for pediatric laparoscopic appendicectomy to reduce the number of surgical trays and single-use items opened per case	Carbon footprint analysis based on average energy and its associated CO_2_e costs to sterilize a single-instrument tray. Conversion factors were obtained from the US Environmental Protection Agency.	33% median reduction in CO_2_e per case
WASTE MANAGEMENT	Leone at al [34] Italy	Optimization of clean and infectious waste segregation in the OR to reduce costs and CO_2_e associated with incineration of biohazardous waste	Method of CO_2_e calculation was not reported	A total of 2809.2 kg of waste was recycled, instead of undergoing incineration, equivalent to saving 1265.04 kg CO_2_e.
	Carmona-Pomada et al. [35] Spain	Multi-level intervention to optimize waste segregation of non-hazardous waste in the OR	Carbon footprint was calculated according to the method of waste disposal. Emission factors were provided by the Catalan Office for Climate Change.	534.6 kg CO_2_e saved per week equivalent to 85% reduction in emissions

## Data Availability

All links to publicly archived datasets analyzed in this study have been included in the manuscript.

## Supplementary Materials

The following supporting information can be downloaded at https://www.mdpi.com/article/10.3390/healthcare14131828/s1, Table S1: Surgical Items included for Calculation of Scope 2 and 3 relative to a cranioplasty pathway.

## Abbreviations

The following abbreviations are used in this manuscript:

CO_2_e	Carbon dioxide equivalents
DEFRA	UK Department for Environment, Food and Rural Affairs
GHG	Greenhouse Gas
HVAC	Heating, ventilation, and cooling
ICE	Inventory of Carbon and Energy
ISO	International Organization for Standardization
LCA	Life cycle assessment
NHS	National Health Service
PEEK	Polyetheretherketone
PEKK	Polyetherketoneketone
PHA	Porous hydroxyapatite
PMMA	Polymethylmethacrylate
PPE	Protective personal equipment
UN-CC-COP	United Nations Climate Change Conference of Parties
