# Supplementary material for: Neurosurgical Theatres’ Carbon Net Efficiency: A Service Improvement Project Conducted via the Oxford Cranioplasty Pathway
Source: Healthcare (Basel). 2026 Jun 24;14(13):1828. doi: 10.3390/healthcare14131828 (PMC13361539; doi:10.3390/healthcare14131828)
Supplement: Supplementary file 1 [file healthcare-14-01828-s001.zip › healthcare-4297027-supplementary.pdf]

CF Calculation Parameters – Cranioplasty

NHS England Greenhouse Gas (GHG) contributions are classified into 3 scopes according to the GHG protocol:

Scope 1 (GHGs directly emitted from and controlled by an organisation):

- Combustion of fossil fuels on site:
  1. Gas boilers (heating) -> if theatres' temperature is maintained via purchased energy (instead of burning fuels onsite) then discard and use only Scope 2 "electricity" category.
- Anaesthetic gases

Scope 2 (GHGs indirectly emitted due to energy purchased):

- Purchased energy in the form of:
  1. Electricity (mostly)
  2. Steam
  3. Heating
  4. Cooling

Scope 3 (Remaining indirect GHGs):

- Pharmaceuticals and chemicals used during procedure
- Medical equipment
- Non-medical equipment
- Water
- Waste disposal

ACTIVITY DATA (SOURCES OF EMISSION) TABLE S1*:**

GHG SCOPE	PARAMETER CATEGORY	PARAMETER SUBCATEGORY/ITEMS	MATERIALS	MEASURE (UNIT)	SOURCE
Scope 1	**Anaesthetic Gases** (volatile)	Gases	Are any of these used?<br>Isoflurane 1.2%<br>Sevoflurane 2.2%<br>Desflurane 6.7%<br>60% Nitrous Oxide	mL/min	From:<br>1. Hospital data obtained at facility.<br>OR<br>2. NHS pharmacy hospital-level electronic data.
Scope 2	**Electricity***	Cooling	N/A	Energy (kWh)	JR Electrical Utilities – metered electricity consumption record
		Ventilation	Type of ventilation system:<br>1. Turbulent mixed airflow<br>OR<br>2. Laminar Air Flow (LAF)<br>OR<br>3.Temperature Controlled Airflow (T_cAF)	Energy (kWh)	JR Electrical Utilities - metered electricity consumption record

		Heating	N/A	Energy (kWh)	JR Electrical Utilities - metered electricity consumption record
		Lighting	N/A	Energy (kWh)	JR Electrical Utilities - metered electricity consumption record
Scope 3	**Medical equipment**	Cranioplasty (device)	Titanium (Ti6AL4V)	Weight (g)	Direct measurement OR manufacturer information
			PEEK	Weight (g)	Direct measurement OR manufacturer information
			Ceramics	Weight (g)	Direct measurement OR manufacturer information
		Cranioplasty (packaging)	Plastic	Weight (g)	Direct measurement OR manufacturer information
		Cranioplasty user manual/surgical guide	Plastic	Weight (g)	Direct measurement OR manufacturer information
		Screws	Titanium	Weight (g)	Direct measurement OR manufacturer information
		Fixtures	Titanium	Weight (g)	Direct measurement OR manufacturer information
		Surgical items used in the procedure (specific to cranioplasty)**		Weight (g)	Direct measurement OR manufacturer information

		PPE (e.g. gowns, gloves etc)		Weight (g)	Direct measurement OR manufacturer information
Scope 3	**Non-medical equipment**	20 mL syringes, gauze etc.			
Scope 3	**Waste disposal***	Dry mixed recyclable waste (clear bag)		Weight (kg)	
	Non-hazardous waste	Domestic waste (black bag)		Weight (kg)	
		Non-infectious offensive waste (yellow/black striped bag)		Weight (kg)	
	Hazardous waste	Infectious waste (orange bag)		Weight (kg)	
		Sharps (yellow lidded yellow box)		Weight (kg)	
		Cytotoxic waste (if any)		Weight (kg)	
		Black box waste (material that is acutely toxic and infectious – if any)		Weight (kg)	
		Anatomical waste (red lidded yellow container – if any)		Weight (kg)	

		Medicinal waste (blue lidded yellow box)		Weight (kg)	
Scope 3	**Non-volatile pharmaceuticals****	Non-volatile anaesthetics and drugs used throughout procedure (e.g. lidocaine, adrenaline etc)		Amount used (mL/kg)	Direct measurement/approximation
	**Non-volatile chemicals****	E.g. Betadine etc.		Amount used (mL)	Direct measurement/approximation

Notes:

*Data considered for calculating the carbon footprint relative to energy consumption and waste disposal of the operating theatre at Oxford University Hospitals have been obtained through primary and secondary data analysis. The latter was obtained from MacNeill et al. [8]

**Materials/constituents of non-volatile pharmaceutical/chemicals and commonly used surgical instruments can be retrieved from Rizan et al. [10]

***Table above needs to be adapted for calculating CF for **unilateral cranioplasty**, **bifrontal cranioplasty** and **cranioplasty after tumour resection**.
